# A retrospective cohort study of infective complications and graft outcomes over 5 years in ABO-incompatible kidney transplant recipients: experience from a tertiary care centre in Saudi Arabia

**DOI:** 10.1097/MS9.0000000000004488

**Published:** 2025-12-04

**Authors:** Bilal Mohsin, Najla Zabani, Nasser Odah, Naief Alhowaiti, Fatmah Yamani, Afnan Al Mutairi, Lama Hefni, Nadeem Shafique Butt, Wael Habhab

**Affiliations:** a Nephrology Section, Department of Internal Medicine, KFSHRC Jeddah, Saudi Arabia; b Department of Family and Community Medicine, King Abdul Aziz Univeristy, Jeddah, Saudi Arabia

**Keywords:** ABOI kidney transplant, covid-19, cytomegalovirus (CMV), graft rejection, UTI

## Abstract

**Background:**

ABO-incompatible (ABOi) kidney transplantation is increasingly utilized to address donor shortages in end-stage kidney disease (ESKD) patients. However, the impact of immunosuppressive regimens on infection risks remains a concern. This study examines the spectrum of infections, associated risk factors, and their influence on graft outcomes over a 5-year period.

**Methods:**

A retrospective cohort study of 24 adult ABOi kidney transplant recipients (2015–2019) was conducted, with follow-up until December 2024. Desensitization included rituximab, plasma exchange (PLEX), and IV immunoglobulin (IVIG). Infections were classified as bacterial, viral, and fungal infections; and the studied outcomes included incidence of infection and their associations with age, gender, comorbidities, graft survival, and graft rejection.

**Results:**

A total of 49 infectious episodes were recorded in 19 patients (79.2%); 5 patients had infection free follow-up of plus 5 years. Urinary tract infections (UTIs) were most common (23/49), followed by COVID-19 (11/49) and Influenza A (7/49). No episode of fungal infection was observed. Infection incidence was highest in females (52.6%), diabetics (47.4%), and patients with prior rejection episodes (10.5%). Kaplan–Meier analysis showed significantly lower infection-free survival in patients with graft rejection (*P* = 0.0086). Despite frequent infections, overall graft survival remained high (91.7%), with no direct statistical association between infections and rejection.

**Conclusion:**

Infections are prevalent in ABOi kidney transplant recipients, particularly in high-risk subgroups, including females, patients with diabetes and prior graft rejection. However, long-term graft survival remains favorable with no association between infections and graft rejection. Optimized immunosuppression and infection surveillance are crucial for improving patient outcomes. Larger multicenter studies are warranted to validate these findings.

## Introduction

Kidney transplantation is a critical therapeutic option for patients with end-stage kidney disease, offering improved quality of life and survival rates compared to dialysis[1]. Although the shortage of compatible donors remains a significant challenge, ABO-incompatible (ABOi) kidney transplantation has emerged as a viable solution to expand the donor pool[2]. Despite its potential benefits, ABOi kidney transplantation is associated with a higher risk of complications, particularly infections, which can impact patient and graft-associated outcomes[3].

Infections are a significant cause of morbidity and mortality in kidney transplant recipients, primarily because immunosuppressive therapy, which is necessary for the prevention of graft rejection[4], increases susceptibility to bacterial, viral, fungal, and parasitic infections[5]. In ABOi kidney transplant recipients, intensified desensitization protocols, which may involve rituximab, intravenous immunoglobulin, and plasmapheresis, further exacerbate the risk of infections[6]

Several studies have highlighted the increased incidence of infectious complications in ABOi renal transplant recipients. For instance, a study conducted in Switzerland found an overall high rate of infections in ABOi kidney transplants compared to ABO-compatible (ABOc) kidney transplants[7] Another study reported a significantly higher rate of viral infections, including cytomegalovirus and polyomavirus, among ABOi recipients[8]. Such infections in ABOi kidney transplant recipients might lead to graft dysfunction and an increased risk of graft failure[9].

Graft dysfunction in ABOi renal transplant recipients can be attributed to several factors, including acute rejection, chronic allograft nephropathy, and infection-associated complications^[10,11]^. Chronic allograft nephropathy, characterized by interstitial fibrosis and tubular atrophy, is often exacerbated by recurrent infections[12]. Viral infection can provoke T-cell-mediated immune responses that trigger the immunological cascade, leading to graft rejection. Although infections pose a significant challenge, existing evidence suggests that long-term graft survival outcomes remain encouraging in ABOi kidney transplant recipients[13].

This study aims to analyze postkidney transplant infective complications and their impact on graft function in ABOi kidney transplant recipients in our patient population, as a single center experience. By evaluating the incidence and outcomes of these complications, we aim to analyze ABOi kidney transplantation as a therapeutic option for ESKD in our patient populations.

## Materials and methods

### Study design and population

This retrospective cohort study included 24 adult ABOi kidney transplants performed at our center between October 31, 2015, and December 30, 2019, with follow-up until December 31, 2024. According to the Declaration of Helenski, the research has been registered with https://www.researchregistry.com (study registry number can be provided as needed). The work has been reported in line with the STROCSS 2025 updated guidelines criteria[14].

The study included adult patients with age more than 14 years, who underwent ABOi kidney transplant in the duration of study. The Exclusion criteria included pediatric ABOi transplants and cases with major surgical or vascular complications requiring surgical re-exploration within 72 hours of kidney transplant. Patients who lost follow up were also excluded from study. Patient confidentiality was maintained using coded identifiers accessible only to authorized team members. The requirement of informed consent from the participants was waived and de identified data was used in this retrospective study.

### Data collection

Patient data, including demographics, comorbid conditions, anti-ABO titers, desensitization protocols, graft function, and infectious and noninfectious complications, were collected from the medical records. Infectious complications were identified with microbiologic cultures, serology, or polymerase chain reaction. Graft loss was defined as return to dialysis, re-transplantation, or death with a functioning graft. Graft rejection was histologically confirmed by ultrasound-guided biopsy using the Banff criteria[15].

### Desensitization protocol

The aim of desensitization protocol was to reduce anti-ABO antibody titers to ≤1:8 in the preoperative time. Rituximab (375 mg/m^2^) was given 2 weeks before desensitization, followed by plasma exchange (PLEX) and intravenous immunoglobulin (IVIG, 0.2 g/kg/session), with a cumulative dose of 1–2 g/kg until anti-ABO titers reached ≤1:8. PLEX dose was calculated via following formula

Plasma Volume (L) = 0.065 × Weight (kg) × (1 − Hematocrit)[16]

### Transplant procedure and immunosuppression

All transplants used living donor grafts, with anastomosis times between 30 and 55 minutes. Induction immunosuppression involved Thymoglobulin (4.5–6 mg/kg) starting intraoperatively (1.5 mg/kg initial dose). Mycophenolate Mofetil, methylprednisolone, and tacrolimus were initiated as per protocol. Methylprednisolone was given at 250 mg on day 0, 125 mg on day 1, tapered by 5 mg daily to 20 mg, and then reduced weekly to a maintenance dose of 5 mg daily. Prophylactic valganciclovir (450 mg/day) was prescribed for 90 days for CMV D +/R + patients and 6 months for CMV D +/R− patients. Co-trimoxazole (80 mg/day) was given for 6–9 months for PCP prophylaxis, initiated when eGFR exceeded 30 mL/min/1.73 m^2^.

**HIGHLIGHTS**

- This study examines infections, risk factors, and their impact on graft outcomes.
- Follow-up duration 5 years.
- Forty-nine infectious episodes noted in 19 patients (79.2%);
- Five patients had infection-free follow-up.
- Most common infections: UTIs (23/49), COVID-19 (11/49), Influenza A (7/49).
- More infections: females (52.6%), diabetics (47.4%), and prior rejection (10.5%).
- No episode of fungal infection.
- Graft survival: 91.7% over follow-up.
- No statistical correlation between infections and rejections.
- Despite infections, long-term graft survival remains high.

### Histological assessment

Renal biopsies were performed when serum creatinine failed to improve within two weeks post-transplant, or for rises >27 μmol/L, proteinuria, hematuria, or suspected rejection. Protocol biopsies were not performed due to patient refusal.

### Outcomes of interest

The studied outcomes included incidence of infections, including UTIs, viral infections (COVID-19, BK virus, CMV), fungal infections, bacteremia; and their association with age, gender, comorbidities, graft rejection and graft failure.

### Statistical analysis

Data were analyzed using SPSS. Continuous variables were expressed as means ± standard deviations (SD) or medians with interquartile ranges (IQR). Categorical variables were presented as frequencies. Fisher’s exact test assessed associations between predictors and outcomes. Kaplan–Meier survival curves were used to evaluate event-free survival from graft failure. A *P*-value ≤ 0.05 was considered statistically significant.

## Results:

Table 1 outlines the demographic and clinical parameters of our study population.

**Table 1:** Demographic and clinical characteristics of ABO incompatible kidney transplant patients (n = 24)

Variable	n (%)	Mean ± SD / Median (IQR)
Donor		
Age (years)		32.7 ± 7.5
Gender	10 (41.7)	
Male	14 (58.3)	
Female		
BMI		24.1 ± 5.7
Recipient		
Age (years)		44.2 ± 13.5
Gender		
Male	11 (45.8)	
13 (54.2)		
BMI		26.0 ± 5.5
Blood group combination		
A+ → B+	1 (4.2)	
A+ → O+	15 (62.5)	
A+ → O-	1 (4.2)	
AB+ → A+	2 (8.3)	
B+ →A+	3 (12.5)	
B+ → O+	2 (8.3)	
Hemoglobin (g/L)		100.5 (93.0-108.5)
WBC (x 10^9^/L)		7.8 ± 3.2
Comorbidities		
Diabetes	9 (37.5)	
Hypertension	20 (83.3)	
Cardiac Disease	1 (4.2)	
COPD	0 (0.0)	
Malignancy	0 (0.0)	
HIV/AIDS, autoimmune disease	0 (0.0)	
Chronic HCV infection	1 (4.2)	
Chronic hepatitis	0 (0.0)	
Hemodialysis	21 (87.5)	
Type of Dialysis		
Peritoneal	1 (4.2)	
Pre-emptive	1 (4.2)	
Type of transplant		
Living related	23 (95.8)	
Living unrelated	1 (4.2)	
Cadaveric	0 (0.0)	
HLA cross match		
Negative	22 (91.6)	
Negative, positive for b cell	1 (4.2)	
Negative with DSA	1 (4.2)	
Pre-TPE sessions		2.0 (2.0-3.0)
Post-TPE sessions		5.0 (3.5-7.0)
IVIG dose (g/kg)		119.5 (80.0-160.0)

COPD, chronic obstructive pulmonary disease; DSA, donor-specific antibodies; WBC, white blood cells;

Mean ± SD or Median (IQR) reported according to distribution of the variable data.

A total of 49 episodes of infections were observed in 19 patients, with 5 patients having infection-free follow-up over 5 years. 59% (*n* = 29) episodes of infections occurred within the first 12 months of transplant while 77.5% (*n* = 38) infection were encountered within the first 18 months after renal transplant. There were 28 episodes of bacterial infections and 21 episodes of viral infections. There was no episode of fungal infection. The mean age of patients experiencing infections is 46 ± 11.1 years, with a median of 51.2 years. 10 of 19 (52.6%) patients were females, and 9 were males. All of these individuals were first kidney transplants. Nine patients had diabetes, while 13 were hypertensive. Two of them had rejection needing intensifying immunosuppressive therapy prior to infective episodes. No patient experienced infections prior to graft rejection.

Thirteen patients had infective episodes with both bacterial and viral infection whether separately or together while six patients had either bacterial or viral infective episode. For the patient with, both, bacterial and viral infections, the mean age 52.3 ± 4.1 years. Nine out of 13 such patients were females, 7 were having diabetes, and 2 had received treatment for graft rejection in the past.

Table 2 shows the distribution of infective episodes in the transplant patients.

**Table 2:** Episodes of infections and their distribution post-transplant (n=49)

CMV	2
COVID	11
BK virus	1
Influenza	7
UTI	23
Bacteremia	5
Fungal infection	0

**Figure 1.**
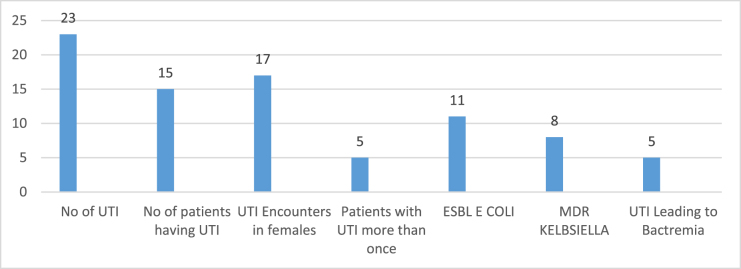
Summary of UTI in patient population.

UTI was the most common infection encountered in the study population, with 23 episodes of infection in 15 patients (Fig. 1). Most of the UTIs (19 episodes) happened in the first 12 months postkidney transplant. The mean age of patients having UTI was 49 ± 3.2 years. Female patients encountered UTI more frequently (17 out of 23 encounters) than males. Five patients had recurrent UTI: 4 females and one male; all 5 of them had diabetes. The most commonly encountered microorganisms were ESBL *Escherichia coli* (*n* = 11), MDR *Klebsiella* (*n* = 8), and *Enterobacter* (*n* = 3). On five occasions, the patient developed bacteremia, needing parenteral antibiotics for more than 1 week.

We encountered COVID-19 infection in 8 patients (*n* = 8), and the total number of encounters with COVID-19 infection was 11, with 3 patients getting infected twice with COVID-19 infection. Five out of eight patients (62.8%) were females, and six (75%) had diabetes. Two of these patients received treatment for graft rejection in the past 6 months. There was no episode of graft loss or mortality during their infective episode with COVID. Five patients had COVID-19 category B, and three had COVID category C symptoms. Two out of three patients with COVID category C needed intensive care monitoring and treatment. Patients needing ICU care developed transient AKI that resolved within 2 weeks of follow-up. As our center protocol, we discontinued Mycophenolate mofetil for patients with COVID-19 category B and C with reduction of target Tacrolimus levels to 3–4 ng/ml with dose increase of prednisolone to 10 mg daily. MMF was resumed 1 week after complete resolution of symptoms.

Influenza was encountered in seven patients. Patients needed hospitalization for 3–5 days and received Oseltamiver for 5 days, followed by complete recovery. We observed cytomegalovirus (CMV) viremia in 8.3 % (*n* = 2) individuals. One patient was diagnosed incidentally on routine for 6 months screening, and the other had GI symptoms needing a workup that revealed CMV viremia. Both incidences occurred between 6 and 18 months after the renal transplant, and both patients had diabetes. We observed BK viremia in one patient who was diagnosed while working up a rising renal profile in the patient. BK virus titers were more than one million copies/mL. Despite modification of immunosuppression, the patient with BK nephropathy progressed to develop chronic kidney disease (CKD) with a functioning graft at the date of the last follow up (55 months). We did not experience fungal infections, malignancy, or mortality in this group.

**Figure 2.**
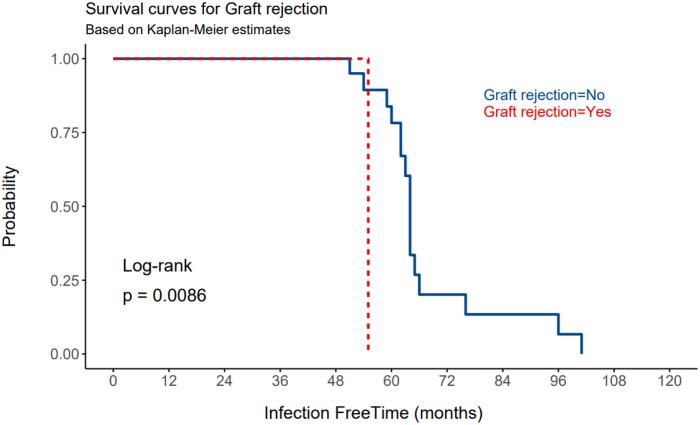
Infection-free aurvival by graft rejection atatus: Kaplan–Meier analysis with log-rank test.

The Kaplan–Meier plot reveals a clear difference in infection-free survival between individuals with and without rejection. (Fig. 2) The group without graft rejection (“Graft rejection = No”) appears to maintain a higher probability of being infection-free over time compared to the group with graft rejection (“Graft rejection = Yes”).

The Log-Rank test yields a *P*-value of 0.0086, less than the conventional threshold of 0.05. This indicates a statistically significant difference in infection-free survival between individuals with and without graft rejection. The plot indicates that the probability of staying infection-free is consistently higher for the group without graft rejection. It also shows higher frequency and clustering of infective events in the early postkidney transplant period.

Our chart review suggests that female gender, diabetes mellitus, and prior history of rejection significantly increase the risk of infections in the renal transplant population.

## Discussion

In our study, we noticed several episodes of bacterial and viral infections in patients undergoing ABOi kidney transplantation. These infections were more frequent in females, patients with diabetes, and patients who experienced graft rejection requiring intensified immunosuppressive therapy. Notably, most of the infections occurred during the first 18 months after kidney transplantation. UTIs were the most frequent infections, followed by COVID-19, influenza A infection, bacteremia, and BK nephropathy. However, despite these infective episodes, the graft survival rate was 91.7% during a mean follow-up period of 64 ± 12.6 months. We noticed two incidences of graft failure, both due to severe antibody-mediated rejection. However, a direct association between infections and long-term graft dysfunction was not observed, supporting the current evidence indicating that infection-related complications can be managed without compromising graft survival^[17,18]^.

A loco-regional study in Saudi Arabia reported similar trends in infection rates among kidney transplant recipients, highlighting the prevalence of bacterial infections such as *E. coli* and *Klebsiella pneumoniae*[19]. These infections were also considered major contributors to graft dysfunction in a Saudi cohort, a finding not consistent with our observations[20]. This discrepancy may stem from the higher immunologic risk in Saudi cohorts, which may underlie the development of graft dysfunction during the postrejection period, rather than infections. Another study in Saudi Arabia also reported that the rate of infectious complications was higher in ABOi kidney transplant recipients than in ABO-compatible kidney transplant recipients and that UTIs were the most common type of infection[21], in agreement with our findings.

Our findings align with international studies reporting increased infection risk in ABOi kidney transplant recipients due to intensified desensitization protocols^[7,9]^. The notable absence of fungal infections in our cohort is in contrast with global studies reporting fungal complications in 5%–10% of the kidney transplant recipients[10]. Our observation might be attributed to regional, environmental and population-based immunologic factors; however, the possibility of under-diagnosis due to limited routine fungal screening and the impact of small sample size cannot be ruled out.

A study from Switzerland also reported higher infection rates in ABOi kidney transplant recipients compared with ABO-compatible kidney transplant recipients, with a particular emphasis on viral infections, such as those caused by CMV and polyomavirus[20]. In the present study, we also observed a significant rate of viral infections, including COVID-19 and influenza A infection, contributing to the complexity of managing these patients. Moreover, although the infection trends in our cohort were comparable to that reported in studies from Europe and North America, we observed relatively lower rates of CMV and BK viremia[22]. This discrepancy may reflect differences in population-based immunologic characteristics, but our small sample size limits detailed analysis in observed differences.

Overall, our findings are consistent with loco-regional and international studies, indicating a higher susceptibility of infections in ABOi kidney transplant recipients, especially in high-risk groups, such as female patients, patients with diabetes, and patients with graft rejection. Despite the prevalence of infectious complications, we observed encouraging short and long term graft outcomes in ABOi kidney transplant recipients.

## Limitations

Despite its valuable insights, our study has several limitations. The small sample size restricted the statistical power to detect subtle associations between infections and graft outcomes. Due to the single-center study design, the study results may not be generalizable to diverse populations with varying immunosuppressive practices. Moreover, the absence of protocol biopsies might have led to the underreporting of subclinical rejection episodes. Finally, we acknowledge the limitations of a retrospective data collection in regards to missing relevant information on infections that might have been diagnosed or treated outside the study institution.

## Conclusion

This study reports infective complications in ABOi kidney transplant recipients, with a higher incidence in females, patients with diabetes and in patients with prior history of rejection. However, despite infective complications, we observed graft survival of 91.7%, over a follow-up of plus 5 years. A small sample size and single-center experience limits our ability to make a consolidative statement but we did not find a significant correlation between infections and graft dysfunction. Hence we suggest a prospective large multicenter trial for further exploration and validation of our findings.

## Data Availability

Data are available upon reasonable request.

## Research registration unique identifying number (UIN)

The UIN is https://www.researchregistry.com/ researchregistry11212
